# Molecular mechanism of crosstalk between immune and metabolic systems in metabolic syndrome

**DOI:** 10.1186/s41232-022-00198-7

**Published:** 2022-05-01

**Authors:** Rumi Hachiya, Miyako Tanaka, Michiko Itoh, Takayoshi Suganami

**Affiliations:** 1 Department of Pediatrics, Keio University School of Medicine, Tokyo, Japan; 2 Department of Pediatrics, Tokyo Dental College Ichikawa General Hospital, Chiba, Japan; 3 Department of Molecular Medicine and Metabolism, Research Institute of Environmental Medicine, Nagoya University, Nagoya, Japan; 4 Department of Immunometabolism, Nagoya University Graduate School of Medicine, Nagoya, Japan; 5 Department of Metabolic Syndrome and Nutritional Science, Research Institute of Environmental Medicine, Nagoya University, Nagoya, Japan; 6 Kanagawa Institute of Industrial Science and Technology, Ebina, Japan

**Keywords:** TLR4, Mincle, Fatty acids, Crown-like structure, Obesity, Metabolic syndrome

## Abstract

Chronic inflammation is currently considered as a molecular basis of metabolic syndrome. Particularly, obesity-induced inflammation in adipose tissue is the origin of chronic inflammation of metabolic syndrome. Adipose tissue contains not only mature adipocytes with large lipid droplets, but also a variety of stromal cells including adipocyte precursors, vascular component cells, immune cells, and fibroblasts. However, crosstalk between those various cell types in adipose tissue in obesity still remains to be fully understood. We focus on two innate immune receptors, Toll-like receptor 4 (TLR4) and macrophage-inducible C-type lectin (Mincle). We provided evidence that adipocyte-derived saturated fatty acids (SFAs) activate macrophage TLR4 signaling pathway, thereby forming a vicious cycle of inflammatory responses during the development of obesity. Intriguingly, the TLR4 signaling pathway is modulated metabolically and epigenetically: SFAs augment TLR4 signaling through the integrated stress response and chromatin remodeling, such as histone methylation, regulates dynamic transcription patterns downstream of TLR4 signaling. Another innate immune receptor Mincle senses cell death, which is a trigger of chronic inflammatory diseases including obesity. Macrophages form a histological structure termed “crown-like structure (CLS)”, in which macrophages surround dead adipocytes to engulf cell debris and residual lipids. Mincle is exclusively expressed in macrophages forming the CLS in obese adipose tissue and regulates adipocyte death-triggered adipose tissue fibrosis. In addition to adipose tissue, we found a structure similar to CLS in the liver of nonalcoholic steatohepatitis (NASH) and the kidney after acute kidney injury. This review article highlights the recent progress of the crosstalk between immune and metabolic systems in metabolic syndrome, with a focus on innate immune receptors.

## Introduction

Evidence has accumulated that proinflammatory cytokines and immune cells contribute to the development and/or progression of metabolic syndrome and its complications, such as diabetes, hepatic steatosis, and atherosclerosis. Thus, chronic inflammation is currently considered as a common molecular basis of those diseases. In chronic inflammation, the parenchymal cells of each organ under metabolic stresses undergo cell death, and stromal cells change their number and cell types, leading to tissue remodeling and organ dysfunction. Innate immune receptors play key roles in intricate cellular interactions among these cell types in metabolic syndrome. This review focuses on obesity-induced inflammation in adipose tissue (adipose tissue inflammation) as the origin of chronic inflammation in metabolic syndrome and the role of two innate immune receptors, namely, Toll-like receptor 4 (TLR4) and macrophage-inducible C-type lectin (Mincle), in the interaction between adipocytes and macrophages. The molecular mechanism of the crosstalk between immune and metabolic systems in metabolic syndrome is discussed. We also highlight chronic inflammation in remote organs, kidney and liver.

## Adipose tissue inflammation as the origin of chronic inflammation in metabolic syndrome

Adipose tissue contains not only mature adipocytes with large lipid droplets but also a variety of stromal cells, including adipocyte precursors, vascular component cells, immune cells, and fibroblasts. During the development of obesity, there must be complex cellular interactions that would affect adipose tissue function, such as lipid storage and adipokine production. This notion was first formulated in 2003 by two American research groups reporting that obesity is associated with macrophage accumulation in adipose tissue [1, 2]. Substantial evidence has pointed to the role of chemokines in recruiting macrophages to adipose tissue, among which the MCP-1 (monocyte chemoattractant protein-1)-CCR2 (C-C chemokine receptor type 2) axis plays a major role [3–6]. Additionally, there are at least two distinct populations of adipose tissue macrophages: CD11c-positive proinflammatory and CD206-positive anti-inflammatory macrophages [7]. However, the crosstalk between cell types in adipose tissue in obesity remains to be fully understood.

## TLR4-regulated inflammatory responses in macrophages

### SFA induces inflammatory responses via TLR4

We have been investigating the molecular mechanisms underlying obesity-induced adipose tissue inflammation. We conceived that there must be crosstalk between adipocytes and macrophages. Using a coculture system composed of adipocytes and macrophages, we found that saturated fatty acids (SFAs) from adipocytes induce the expression of proinflammatory cytokines, such as TNFα (tumor necrosis factor alpha) and IL-6 (interleukin-6) through TLR4 in macrophages. In turn, such macrophage-derived proinflammatory cytokines act on adipocytes to activate lipolysis. Thus, we provided evidence that adipocytes and macrophages interact through secreted factors, thereby forming a vicious cycle of inflammatory responses during the development of obesity [8, 9]. Moreover, TLR4 is crucial for the phenotypic change of macrophages to become CD11c-positive [10, 11]. TLR4 deficiency mitigates obesity-induced adipose tissue inflammation and systemic insulin resistance in mice [12–14].

In the innate immune response, bacterial endotoxin lipopolysaccharide (LPS) is an authentic ligand for TLR4. As addressed above, substantial evidence has demonstrated the role of TLR4 in obesity-induced adipose tissue inflammation [12–14]. Various reports have been made on the mechanism by which adipocyte-derived SFAs, such as palmitate and stearate, control inflammation associated with obesity. Mainly based on in vitro evidence, palmitate is thought to activate TLR4 signaling pathways in macrophages [9, 15]. Recently, Lancaster et al. reported that palmitate is actually not a direct TLR4 agonist and that TLR4 reprograms cellular metabolism to induce SFA-mediated inflammatory responses [16], although the precise molecular mechanisms are still controversial. Meanwhile, polyunsaturated fatty acids, such as eicosapentaenoic acid and docosahexaenoic acid, counteract the effects of SFAs [15, 17]. Thus, the quantity and quality of adipocyte-stored lipids should affect the paracrine loop involving mature adipocytes and macrophages, thereby regulating adipose tissue inflammation [18, 19].

### SFA-induced integrated stress response augments TLR4 signaling

**Fig. 1:**
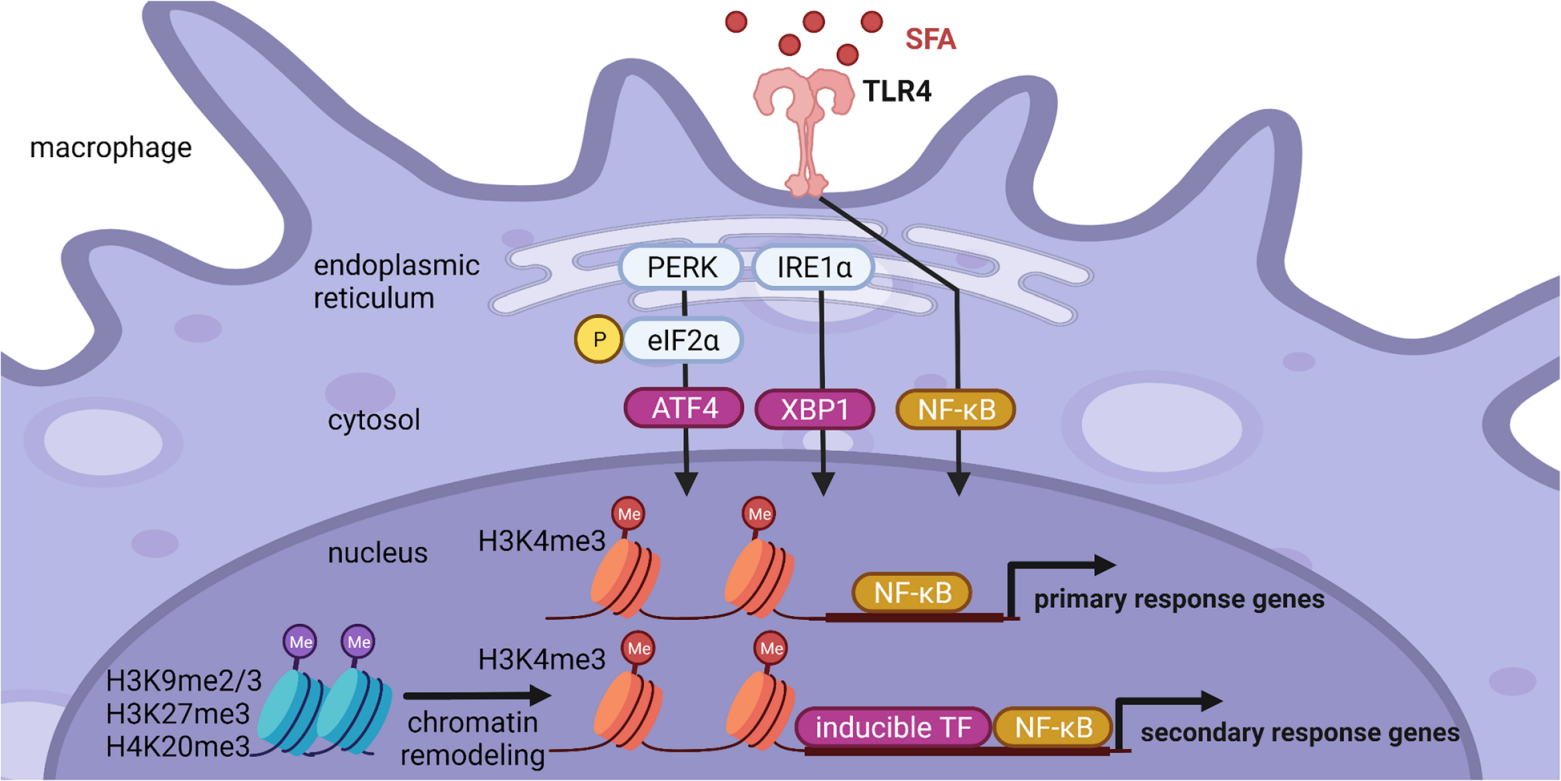
Potential mechanism of metabolic and epigenetic regulation of TLR4 signaling. ER is an interface between immune and metabolic systems. PERK and IRE1α, along with their downstream effectors, ATF4 and XBP1, respectively, are involved in SFA-induced metabolic inflammatory responses. TLR4 signal–mediated proinflammatory cytokines are divided into the following two groups. Primary response genes are rapidly upregulated in response to stimuli, while secondary response genes are induced later. Accumulating evidence has highlighted the significant role of chromatin remodeling in the regulation of these upon TLR4 activation. Among others, covalent modifications at histones H3 and H4 have been shown to play a key role in regulating chromatin assembly and the recruitment of inducible transcription factors, suggesting the epigenetic regulation of TLR4 signaling. Created with BioRender.com

Although innate immune receptors, such as TLR4, play important roles in infectious and non-infectious diseases, inflammatory responses induced by SFAs appear different in their time course and upregulated genes from those induced by endotoxins. Indeed, SFAs induce proinflammatory cytokine expression even in TLR4-deficient macrophages [20]. These observations led us to speculate that metabolic pathways are involved in SFA-induced proinflammatory cytokine expression in macrophages. By means of transcriptome analysis, we focused on the integrated stress response (ISR) [20], which is commonly activated under endoplasmic reticulum (ER) stress, hypoxic stress, and oxidative stress and is implicated in the pathogenesis of metabolic syndrome [21]. The ISR includes the phosphorylation of eukaryotic initiation factor-2α (eIF2α) and subsequent activation of activating transcription factor 4 (ATF4). We have demonstrated that SFAs induce proinflammatory cytokine expression through the ISR in which ATF4 directly binds to and activates the *IL-6* promoter [20]. ATF4 also enhances the nuclear localization of NF-κB. Thus, the ISR augments TLR4-mediated inflammatory responses. With regard to the SFA-induced activation of the ISR, previous studies pointed to the involvement of ER stress [22–25]. SFAs are incorporated through fatty acid transporters, such as CD36, metabolized to phospholipids and diacylglycerol, and then accumulated in the ER [22, 23]. Changes in the lipid composition of the ER membrane activate unfolded protein responses, including the ISR [24, 25]. It was also reported that another branch of unfolded protein responses, i.e., the inositol-requiring enzyme 1α (IRE1α)-X-box binding protein 1 (XBP1) pathway, is involved in TLR4-mediated proinflammatory cytokine expression [26]. Moreover, our recent data show that macrophages rely on extracellular serine, a major nonessential amino acid, to suppress aberrant cytokine production upon TLR4 stimulation [27]. Collectively, these findings highlight the crosstalk between cellular metabolism and inflammatory signals in immune cells, suggesting that metabolic conditions modify proinflammatory responses in immune cells (Fig. 1).

### Epigenetic regulation of TLR4 signaling

The TLR4 signaling pathway in macrophages has been intensively investigated. In principle, proinflammatory cytokines downstream of TLR4 are divided into the following two groups. Primary response genes, including TNFα, are rapidly upregulated in response to stimuli, while secondary response genes, including IL-6, are induced later [28–30]. The transcription factor NF-κB plays a critical role in the induction of primary response genes, whereas other inducible transcription factors, such as C/EBPs (CCAAT/enhancer binding proteins), are required in the induction of secondary response genes. Accumulating evidence has highlighted the significant role of chromatin remodeling in the regulation of proinflammatory cytokine expression [28–30]. Among others, covalent modifications at histones H3 and H4 have been shown to play a key role in regulating chromatin assembly and the recruitment of inducible transcription factors [31].

Histone H3 lysine 4 trimethylation (H3K4me3) positively regulates the induction of proinflammatory cytokines [32, 33]. For instance, the macrophage-specific deletion of KMT2A (also called MLL1) results in decreased expression of some primary and secondary response genes, along with reduced H3K4me3 levels [34]. Moreover, repressive histone modifications, such as H3K9me2/3, H3K27me3, and H4K20me3, are reported as an important regulatory mechanism of inflammation [31]. Recently, we demonstrated that H3K9 methyltransferase Setdb1 (SET domain, bifurcated 1) suppresses TLR4-mediated proinflammatory cytokine expression in macrophages in vivo and in vitro. Our data showed that H3K9 methyltransferase activity is required for the anti-inflammatory role of Setdb1 [35]. The role of Setdb1 in SFA-induced inflammation remains to be determined, whereas another H3K9 methyltransferase, G9a, has been recently reported as a mediator of SFA-induced M1 macrophage polarization through negatively regulating CD36 by H3K9 methyltransferase activity [36]. Thus, it is interesting to investigate the involvement of histone modifications in SFA-induced TLR4 activation in metabolic syndrome. H3K9 methylation was once considered stable and irreversible, but we and others have reported that dynamic changes in H3K9 methylation occur in the promoter region of certain proinflammatory genes in response to inflammatory stimuli [35, 37, 38]. For instance, Villeneuve et al*.* reported that the H3K9 methylation levels in the promoter region of proinflammatory cytokines decrease under hyperglycemic conditions in vascular smooth muscle cells, leading to increased proinflammatory cytokine expression [37]. van Essen et al*.* also reported that Aof1, an H3K9 demethylase, is recruited to the promoter region of proinflammatory cytokines under LPS treatment in dendritic cells, decreasing the H3K9 methylation levels and increasing the recruitment of NF-κB [38]. As for other repressive histone modifications, KDM6A, an H3K27 demethylase, accelerates proinflammatory responses in LPS-stimulated macrophages [39, 40]. Moreover, H4K20 trimethylation/demethylation is required to induce proinflammatory gene expression [41]. Future work should investigate how innate immune signaling pathways target specific chromatin modifiers to regulate gene-specific epigenetic mechanisms. Based on these observations, potential mechanisms of epigenetic regulation of TLR4 signaling are shown in Fig. 1.

## Mincle-regulated tissue remodeling and CLS

### Mincle mediates cell death-triggered inflammation

**Fig. 2:**
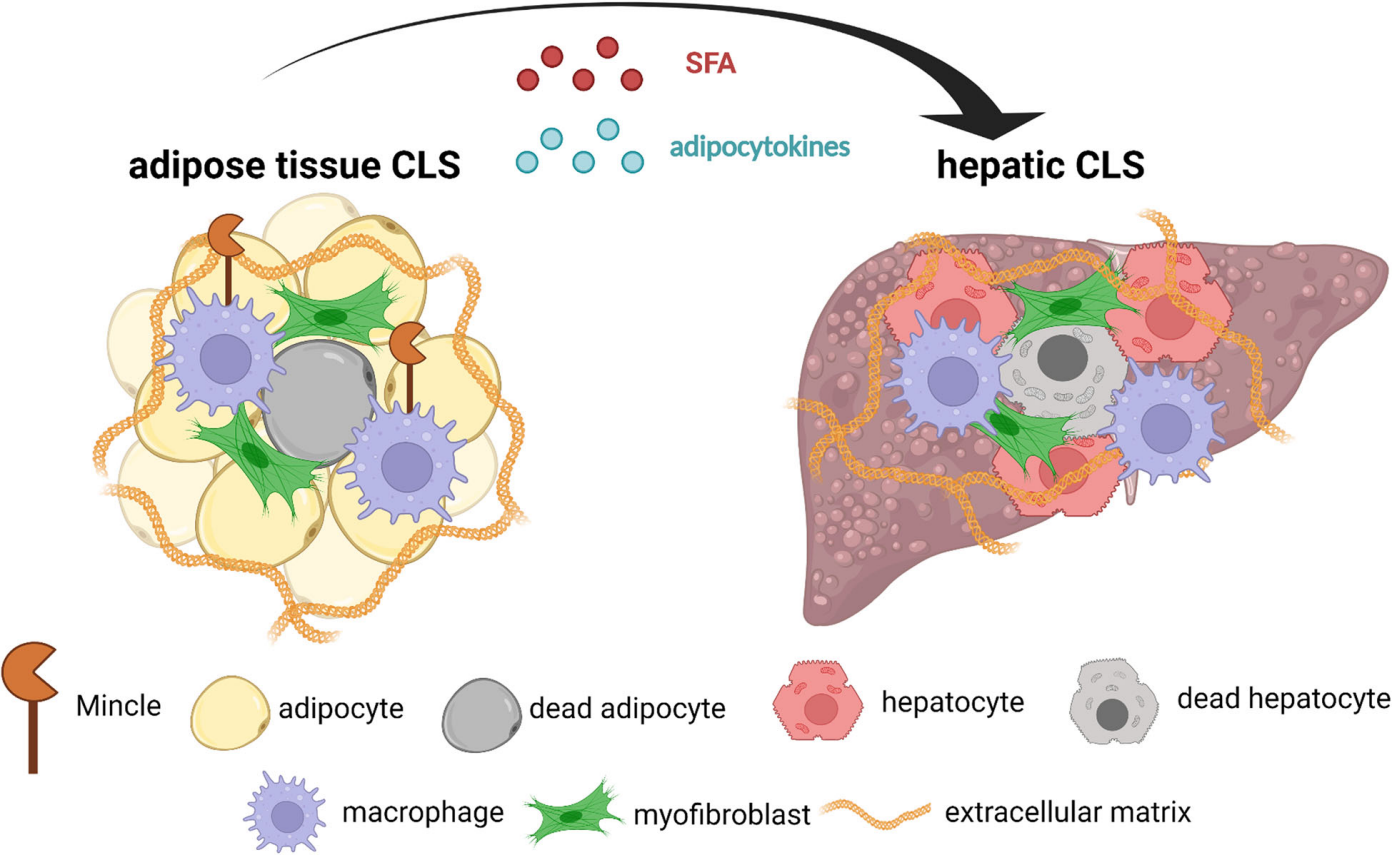
CLS is a hallmark of obesity-induced chronic inflammation. Obesity induces inflammation and fibrosis in adipose tissue and the liver. Chronic inflammation leads to a characteristic histological structure termed “crown-like structure (CLS)” in which macrophages surround dead adipocytes, resulting in fibrosis. An innate immune receptor Mincle functions as a cell death sensor, which is selectively upregulated in the macrophages that constitute CLS in obese adipose tissue. Mincle regulates adipocyte death-triggered fibrogenesis and controls lipid storage function of adipose tissue, thereby affecting ectopic lipid accumulation in remote organs such as the liver. In nonalcoholic steatohepatitis, there is a structure similar to CLS in which macrophages surround dead hepatocytes with excessive lipids. Created with BioRender.com

In addition to TLR4, accumulating evidence has suggested a role for other innate immune receptors in the pathogenesis of adipose tissue inflammation. Among others, we focused on Mincle, a type II membrane protein in macrophages whose expression is markedly induced by the LPS treatment [42]. Mincle functions as an innate immune receptor that recognizes *Mycobacterium tuberculosis* and certain types of pathogenic fungi [43–45]. Moreover, Mincle senses dead cells in vitro, suggesting a role for it in sterile inflammation [46]. However, Mincle’s in vivo role as a cell death sensor remains to be elucidated. Cell death is a trigger of chronic inflammatory diseases, including obesity. Indeed, adipocytes undergo cell death due to metabolic stress during the development of obesity [47]. Interestingly, macrophages form a unique histological assembly known as “crown-like structure (CLS)” in which macrophages surround dead adipocytes to engulf cell debris and residual lipids [48] (Fig. 2). Since these macrophages possess proinflammatory properties, the CLS is a hallmark of obesity-induced adipose tissue inflammation. Consistently, the number of CLS is positively correlated with systemic insulin resistance in experimental animal models and human subjects. The CLS comprises bone marrow-derived CD11c-positive macrophages and Mincle is exclusively expressed in macrophages forming the CLS in obese adipose tissue, under the control of TLR4 signaling [10]. Thus, it is conceivable that CLS-forming macrophages sense dead adipocytes, which leads to their phenotypic change, thereby activating proinflammatory programs. We provided evidence that Mincle plays a key role in this process [49] (Fig. 2).

We have demonstrated that Mincle deficiency does not affect body weight gain in diet-induced obese mice [49]. Notably, adipose tissue weight is significantly higher in Mincle-deficient mice compared to that in wild-type mice, whereas liver weight is lower in Mincle-deficient mice. These findings suggest that Mincle regulates the distribution of lipid storage in the body. Within adipose tissue, Mincle deficiency markedly inhibits obesity-induced CLS formation and interstitial fibrosis without affecting the number of infiltrating macrophages. Our data suggest that Mincle activation in macrophages induces the expression of transforming growth factor beta (TGFβ), a master regulator of fibrogenesis, which activates surrounding fibroblasts and induces collagen production, resulting in adipose tissue fibrosis.

The primary function of adipose tissue is to store excess energy as triglyceride. It is well known that the endocrine (insulin) and sympathetic nervous systems regulate lipogenesis and lipolysis, respectively [50]. Additionally, recent evidence indicates the involvement of chronic inflammation in this process. Indeed, chronic inflammation and resulting interstitial fibrosis contribute to “unhealthy” adipose tissue expansion in obesity, in which adipocyte expandability and the lipid storage function are disturbed [51]. In this regard, Pasarica et al*.* reported that expression of collagen VI, a highly enriched extracellular matrix component of adipose tissue, is strongly associated with fat mass and inflammatory markers in obese patients [52]. Kahn et al*.* showed that deficiency of collagen VI in mice results in the uninhibited expansion of individual adipocytes and paradoxical improvement of hepatic steatosis [53]. Collectively, these observations suggest the role of obesity-induced adipose tissue inflammation in ectopic lipid accumulation [19]. In line with this notion, our data indicate that Mincle regulates adipocyte death-triggered adipose tissue inflammation and fibrosis, which controls the lipid storage function of adipose tissue, thereby affecting ectopic lipid accumulation in remote organs (Fig.2) [49].

### Identification of cell death-derived endogenous Mincle ligands

To date, innate immune receptors, including TLR4 and Mincle have been implicated in the pathogenesis of sterile inflammation. Damage-associated molecular patterns (DAMPs), secreted or released from dead/dying cells, act on innate immune receptors to induce proinflammatory responses in immune cells [54, 55]. However, only a limited number of DAMPs have been found in in vivo disease models. Recently, we successfully identified an endogenous Mincle ligand in a mouse model of acute kidney injury [56]. Mincle deficiency protects against tubular death-triggered inflammation after renal ischemia-reperfusion injury. Similar to adipose tissue in obesity, Mincle expression is localized to macrophages surrounding necrotic tubules in the injured kidney. Because most previously known Mincle ligands are lipids [57], we screened for endogenous Mincle ligands using lipids extracted from the injured kidney. By means of nontargeted lipidomics analysis, we identified β-glucosylceramide as an endogenous Mincle ligand. Intriguingly, the ligand activity of β-glucosylceramide is relatively weak by itself relative to the lipid fraction extracted from the injured kidney. We finally found that free cholesterol markedly enhances the ligand activity of β-glucosylceramide. Histologically, free cholesterol is accumulated in necrotic tubules within a structure similar to CLS in the cortico-medullary junction area of the injured kidney. This finding is reminiscent of a previous report showing that there is free cholesterol or cholesterol crystals within the CLS in obese adipose tissue [58]. Besides proinflammatory cytokine production, our data have revealed a novel function of Mincle, whereby it suppresses the clearance of dead cells [56]. Accordingly, Mincle activation is exerted to maintain the CLS, which is supposed to sustain inflammation in regions in close proximity.

### CLS-mediated liver fibrosis in nonalcoholic steatohepatitis

We found a histological structure similar to the CLS in animal NASH models and human NASH, where dead hepatocytes with excessive lipids are surrounded by CD11c-positive macrophages and activated fibroblasts (myofibroblasts) [59–61] (Fig.2). In the pathogenesis of NASH, hepatic CLS is crucial as the interface between metabolism and immunity. Unlike adipose tissue CLS, hepatic CLS does not express Mincle [49, 56]. Therefore, it is necessary to identify the innate immune sensor in NASH. Lipid accumulation in the liver is a feature of metabolic syndrome. In particular, NASH, characterized by chronic inflammation and interstitial fibrosis, predisposes patients to cirrhosis and hepatocellular carcinoma. Currently, the “multiple parallel hits” hypothesis has been proposed as the molecular pathogenesis of NASH in which combination of metabolic stresses (e.g*.*, lipid accumulation and insulin resistance) and inflammatory stimuli (e.g., proinflammatory cytokines and endotoxins) gives rise to the progression to NASH from simple steatosis [62]. However, it is still unknown what triggers chronic inflammation and fibrosis in this process. Of note, CLS formation precedes the development of liver fibrosis. In clinical settings, CLS formation is observed in patients with simple hepatic steatosis and NASH, but not in patients with chronic viral hepatitis [59]. Thus, CLS in the liver promotes pericellular fibrosis, a histological feature of NASH. Collectively, adipose tissue (obesity), the kidney (acute kidney injury), and the liver (NASH) exhibit similar unique microenvironments in which dead parenchymal cells surrounded by macrophages serve as a driving engine of chronic inflammatory responses. On the other side, distinct innate immune receptors are responsible for each disease. Identifying the role of innate immune receptors and their endogenous ligands in various chronic inflammatory diseases will be of interest to compare common and disease-specific mechanisms.

## Conclusions

**Fig. 3:**
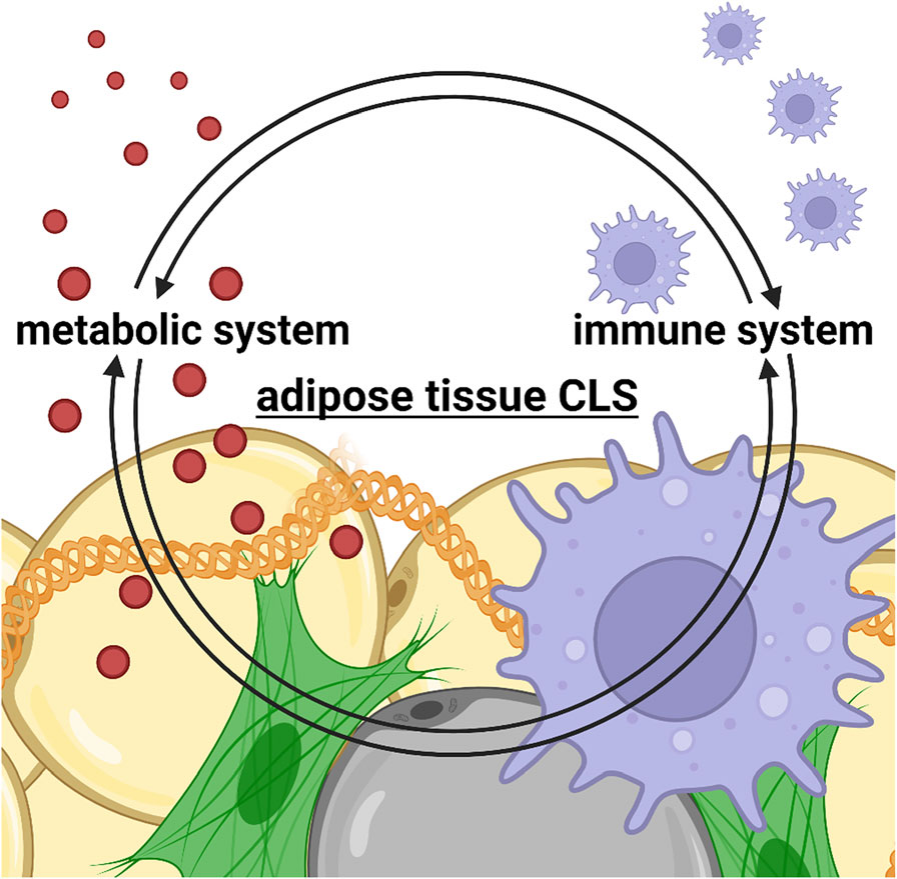
An interface between immune and metabolic systems: adipose tissue CLS. Immune cells infiltrate adipose tissue in response to body weight gain, induce chronic inflammation in adipose tissue, and affect the functions of remote organs, such as the liver, through aberrant adipokine production. In this regard, the CLS serves as a driving engine to induce metabolic inflammatory responses in obese adipose tissue (adipose tissue CLS). Intriguingly, a similar structure occurs in the liver in the progression from simple hepatic steatosis to NASH (hepatic CLS). Thus, the considerable attention to the interface between immune and metabolic systems has led to the emerging concept of “immunometabolism.” Created with BioRender.com

Based on the accumulating evidence during the past two decades, metabolic syndrome is currently considered a chronic inflammatory disease. Particularly, immune cells infiltrate adipose tissue in response to body weight gain, induce chronic inflammation in adipose tissue, and affect the functions of remote organs, such as the liver, through aberrant adipokine production [18, 19]. On the other hand, considerable evidence has identified a role for metabolic reprogramming in the activation and differentiation of immune cells. Thus, substantial attention to the interface between the immune and metabolic systems has led to the emerging concept of “immunometabolism” (Fig. 3) [63, 64]. This review discussed that innate immune receptors, such as TLR4 and Mincle, are involved in interactions between parenchymal cells and macrophages, and cellular metabolism modifies immune cell function, thereby affecting the systemic metabolic homeostasis. Additionally, we discussed recent advances in our understanding of how parenchymal cells and macrophages interact to induce chronic inflammation, with a focus on a unique microenvironment, known as the CLS, composed of dead parenchymal cells and macrophages. In the future, the development of novel therapeutic strategies for metabolic syndrome targeting chronic inflammation is expected.

## Data Availability

N/A.

## Abbreviations

ATF4: Activating transcription factor 4
CCR2: C-C chemokine receptor type 2
C/EBPs: CCAAT/enhancer binding proteins
CLS: Crown-like structure
DAMPs: Damage-associated molecular patterns
eIF2α: Eukaryotic initiation factor-2α
ER: Endoplasmic reticulum
FFA: Free fatty acid
IL-6: Interleukin-6
ISR: Integrated stress response
IRE1α: Inositol-requiring enzyme 1α
LPS: Lipopolysaccharide
Mincle: Macrophage-inducible C-type lectin
MCP-1: Monocyte chemoattractant protein-1
NASH: Nonalcoholic steatohepatitis
Setdb1: SET domain, bifurcated 1
SFA: Saturated fatty acid
TGFβ: Transforming growth factor β
TNFα: Tumor necrosis factor alpha
TLR4: Toll-like receptor 4
XBP1: X-box binding protein
